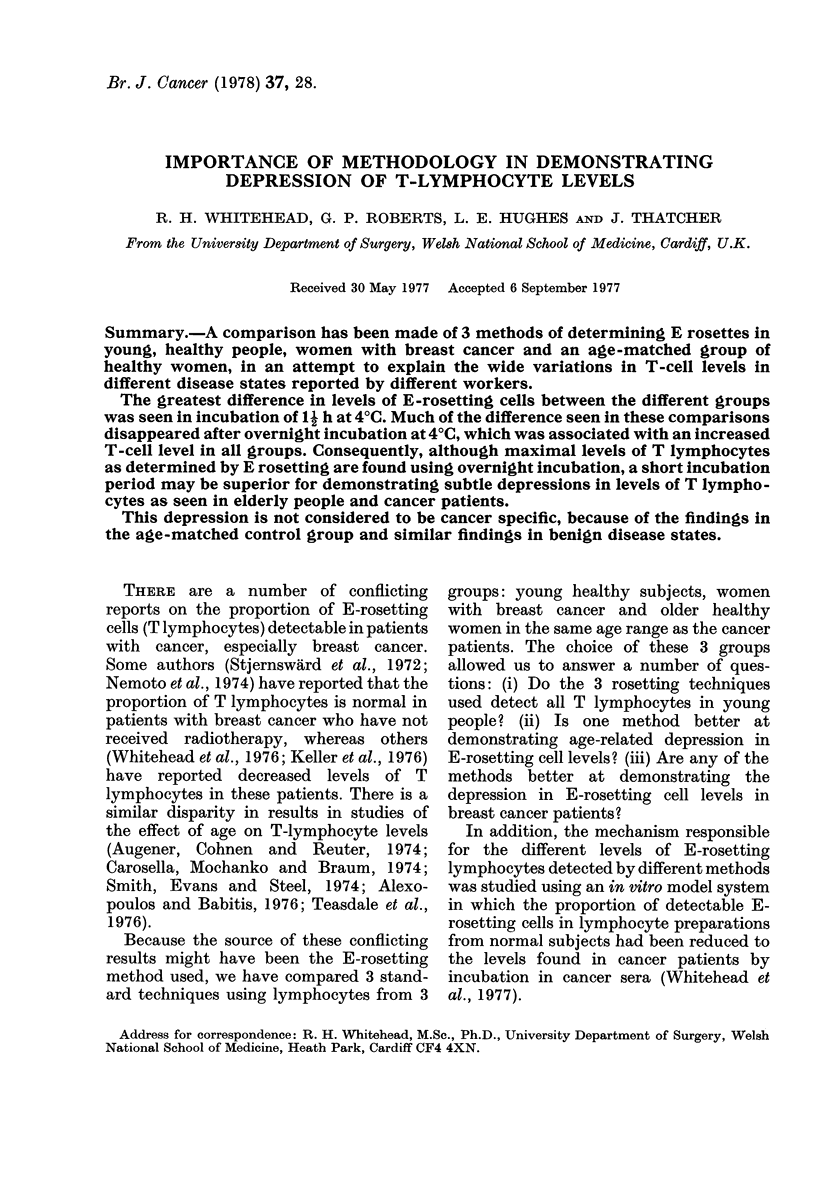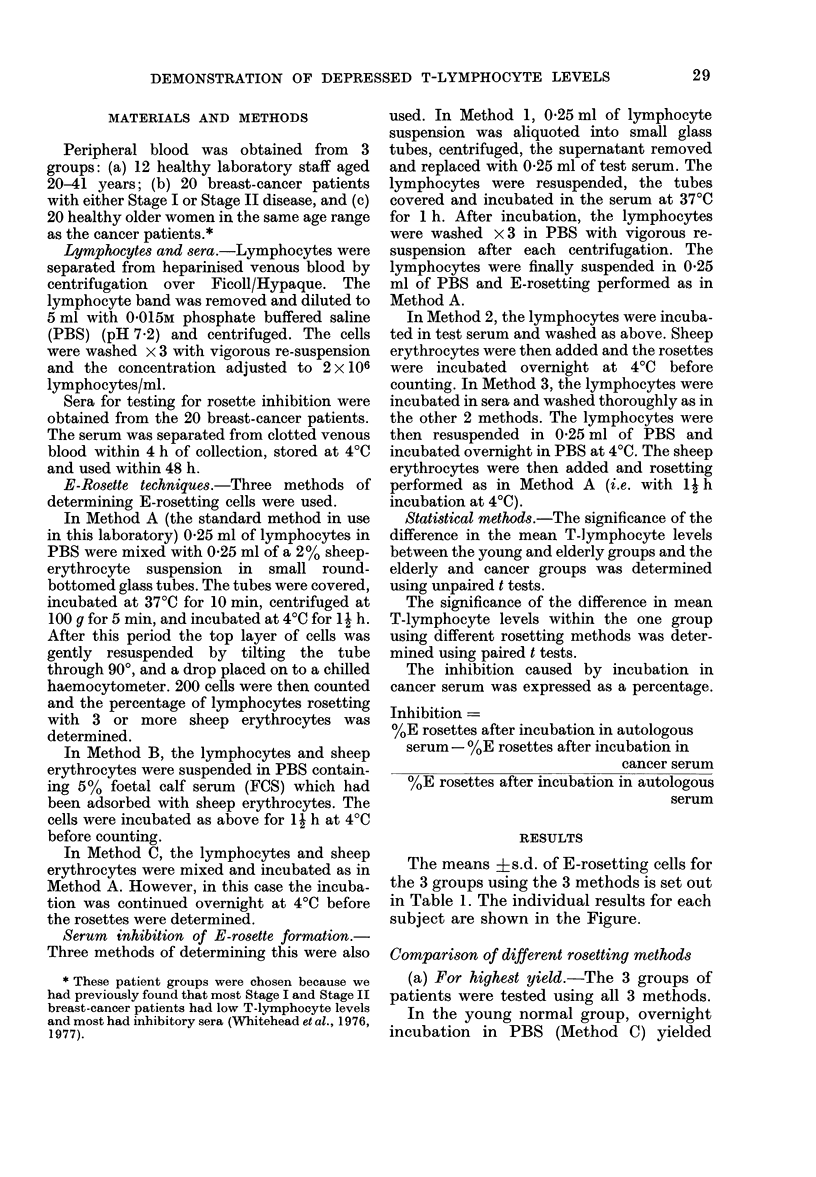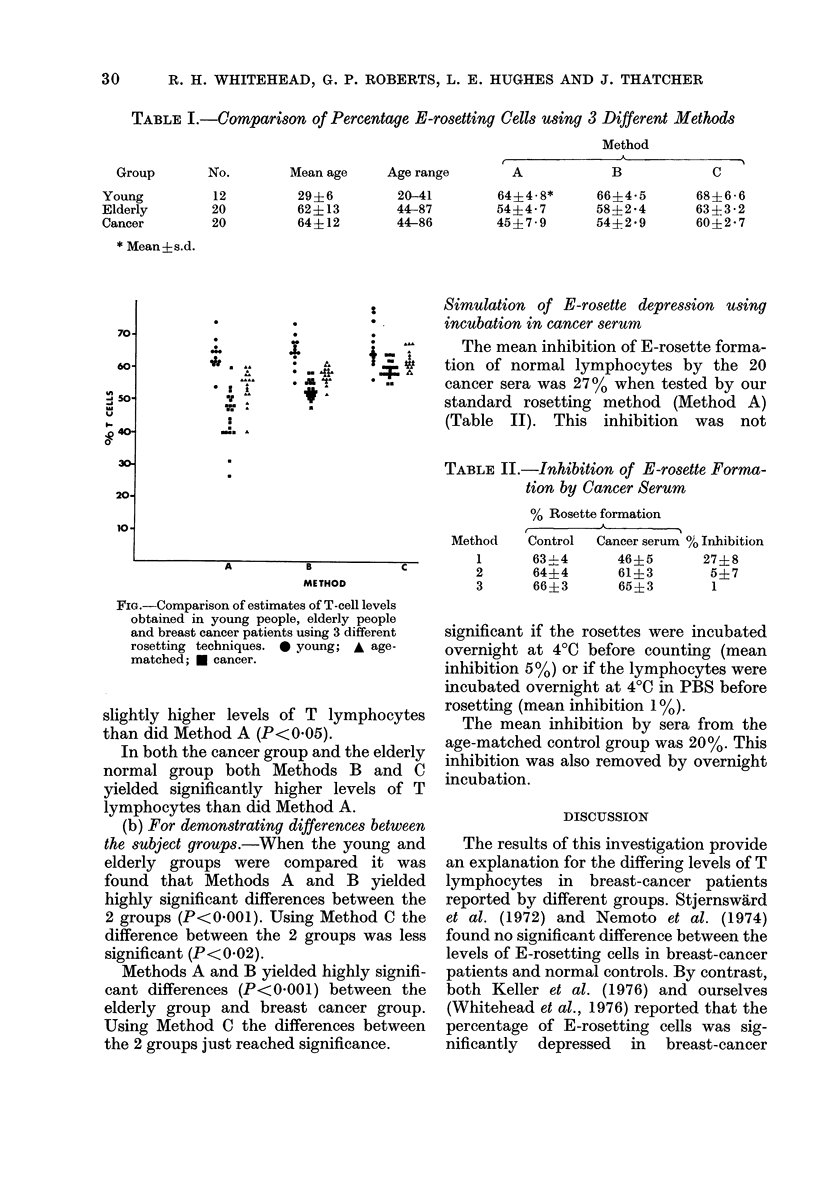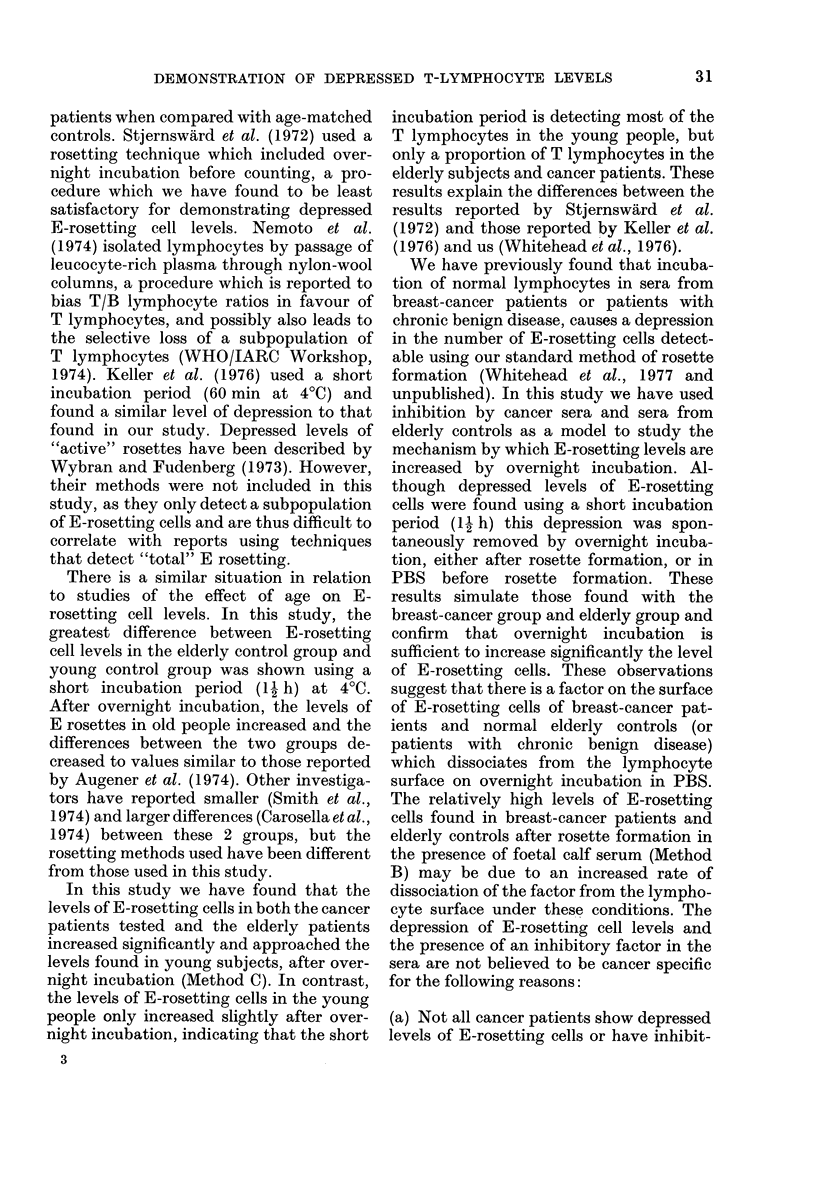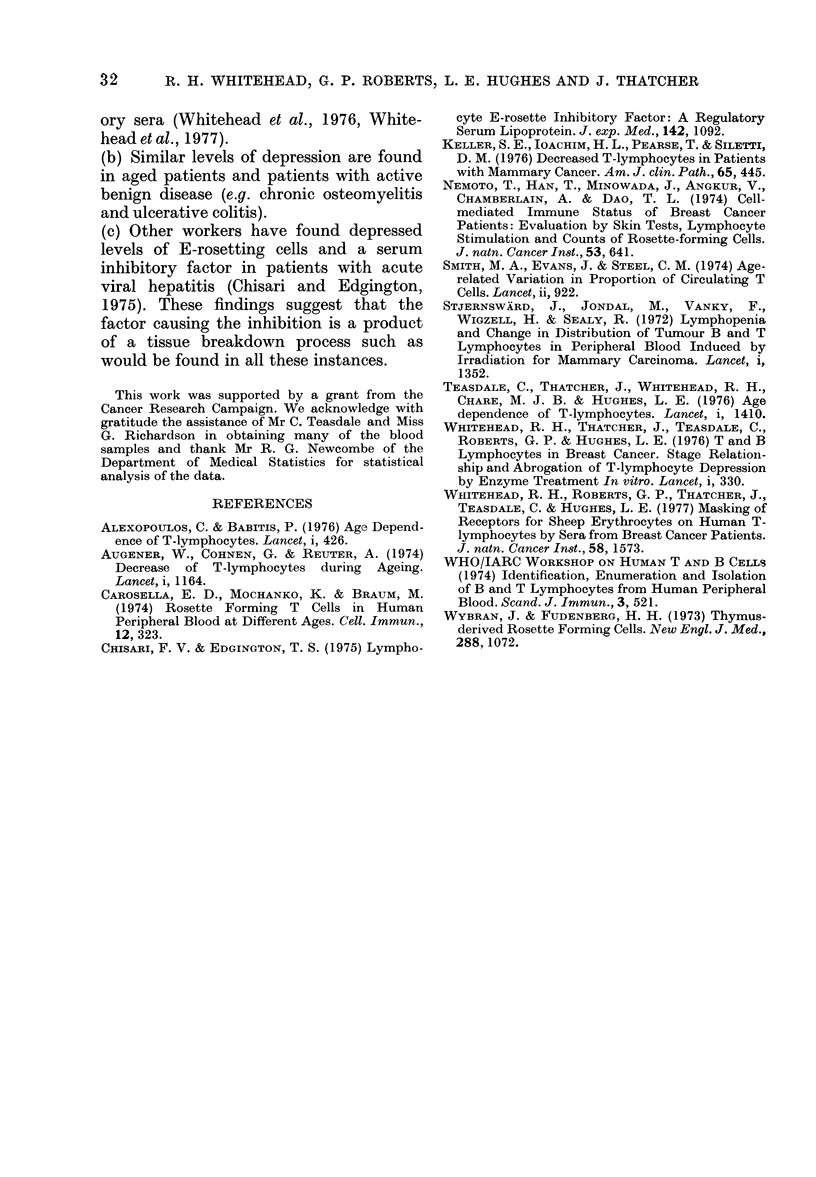# Importance of methodology in demonstrating depression of T-lymphocyte levels.

**DOI:** 10.1038/bjc.1978.4

**Published:** 1978-01

**Authors:** R. H. Whitehead, G. P. Roberts, L. E. Hughes, J. Thatcher

## Abstract

A comparison has been made of 3 methods of determining E rosettes in young, healthy people, women with breast cancer and an age-matched group of healthy women, in an attempt to explain the wide variations in T-cell levels in different disease states reported by different workers. The greatest difference in levels of E-rosetting cells between the different groups was seen in incubation of 1 1/2 h at 4 degrees C. Much of the difference seen in these comparisons disappeared after overnight incubation at 4 degrees C, which was associated with an increased T-cell level in all groups. Consequently, although maximal levels of T lymphocytes as determined by E rosetting are found using overnight incubation, a short incubation period may be superior for demonstrating subtle depressions in levels of T lymphocytes as seen in elderly people and cancer patients. This depression is not considered to be cancer specific, because of the findings in the age-matched control group and similar findings in benign disease states.


*Br. J. Cancer* (1978) **37**, 28.

### IMPORTANCE OF METHODOLOGY IN DEMONSTRATING DEPRESSION OF T-LYMPHOCYTE LEVELS

R. H. WHITEHEAD, G. P. ROBERTS, L. E. HUGHES AND J. THATCHER

*From the University Department of Surgery, Welsh National School of Medicine, Cardiff, U.K.*

Received 30 May 1977 Accepted 6 September 1977

**Summary.—A comparison has been made of 3 methods of determining E rosettes in young, healthy people, women with breast cancer and an age-matched group of healthy women, in an attempt to explain the wide variations in T-cell levels in different disease states reported by different workers.**

**The greatest difference in levels of E-rosetting cells between the different groups was seen in incubation of 1½ h at 4°C. Much of the difference seen in these comparisons disappeared after overnight incubation at 4°C, which was associated with an increased T-cell level in all groups. Consequently, although maximal levels of T lymphocytes as determined by E rosetting are found using overnight incubation, a short incubation period may be superior for demonstrating subtle depressions in levels of T lymphocytes as seen in elderly people and cancer patients.**

**This depression is not considered to be cancer specific, because of the findings in the age-matched control group and similar findings in benign disease states.**

THERE are a number of conflicting reports on the proportion of E-rosetting cells (T lymphocytes) detectable in patients with cancer, especially breast cancer. Some authors (Stjernswärd *et al.*, 1972; Nemoto *et al.*, 1974) have reported that the proportion of T lymphocytes is normal in patients with breast cancer who have not received radiotherapy, whereas others (Whitehead *et al.*, 1976; Keller *et al.*, 1976) have reported decreased levels of T lymphocytes in these patients. There is a similar disparity in results in studies of the effect of age on T-lymphocyte levels (Augener, Cohnen and Reuter, 1974; Carosella, Mochanko and Braum, 1974; Smith, Evans and Steel, 1974; Alexopoulos and Babitis, 1976; Teasdale *et al.*, 1976).

Because the source of these conflicting results might have been the E-rosetting method used, we have compared 3 standard techniques using lymphocytes from 3 groups: young healthy subjects, women with breast cancer and older healthy women in the same age range as the cancer patients. The choice of these 3 groups allowed us to answer a number of questions: (i) Do the 3 rosetting techniques used detect all T lymphocytes in young people? (ii) Is one method better at demonstrating age-related depression in E-rosetting cell levels? (iii) Are any of the methods better at demonstrating the depression in E-rosetting cell levels in breast cancer patients?

In addition, the mechanism responsible for the different levels of E-rosetting lymphocytes detected by different methods was studied using an *in vitro* model system in which the proportion of detectable E-rosetting cells in lymphocyte preparations from normal subjects had been reduced to the levels found in cancer patients by incubation in cancer sera (Whitehead *et al.*, 1977).

Address for correspondence: R. H. Whitehead, M.Sc., Ph.D., University Department of Surgery, Welsh National School of Medicine, Heath Park, Cardiff CF4 4XN.

### MATERIALS AND METHODS

Peripheral blood was obtained from 3 groups: (a) 12 healthy laboratory staff aged 20–41 years; (b) 20 breast-cancer patients with either Stage I or Stage II disease, and (c) 20 healthy older women in the same age range as the cancer patients.*

*Lymphocytes and sera.*—Lymphocytes were separated from heparinised venous blood by centrifugation over Ficoll/Hypaque. The lymphocyte band was removed and diluted to 5 ml with 0·015M phosphate buffered saline (PBS) (pH 7·2) and centrifuged. The cells were washed ×3 with vigorous re-suspension and the concentration adjusted to $2\times10^6$ lymphocytes/ml.

Sera for testing for rosette inhibition were obtained from the 20 breast-cancer patients. The serum was separated from clotted venous blood within 4 h of collection, stored at 4°C and used within 48 h.

*E-Rosette techniques.*—Three methods of determining E-rosetting cells were used.

In Method A (the standard method in use in this laboratory) 0·25 ml of lymphocytes in PBS were mixed with 0·25 ml of a 2% sheep-erythrocyte suspension in small round-bottomed glass tubes. The tubes were covered, incubated at 37°C for 10 min, centrifuged at 100 *g* for 5 min, and incubated at 4°C for 1½ h. After this period the top layer of cells was gently resuspended by tilting the tube through 90°, and a drop placed on to a chilled haemocytometer. 200 cells were then counted and the percentage of lymphocytes rosetting with 3 or more sheep erythrocytes was determined.

In Method B, the lymphocytes and sheep erythrocytes were suspended in PBS containing 5% foetal calf serum (FCS) which had been adsorbed with sheep erythrocytes. The cells were incubated as above for 1½ h at 4°C before counting.

In Method C, the lymphocytes and sheep erythrocytes were mixed and incubated as in Method A. However, in this case the incubation was continued overnight at 4°C before the rosettes were determined.

*Serum inhibition of E-rosette formation.*—Three methods of determining this were also used. In Method 1, 0·25 ml of lymphocyte suspension was aliquoted into small glass tubes, centrifuged, the supernatant removed and replaced with 0·25 ml of test serum. The lymphocytes were resuspended, the tubes covered and incubated in the serum at 37°C for 1 h. After incubation, the lymphocytes were washed ×3 in PBS with vigorous re-suspension after each centrifugation. The lymphocytes were finally suspended in 0·25 ml of PBS and E-rosetting performed as in Method A.

In Method 2, the lymphocytes were incubated in test serum and washed as above. Sheep erythrocytes were then added and the rosettes were incubated overnight at 4°C before counting. In Method 3, the lymphocytes were incubated in sera and washed thoroughly as in the other 2 methods. The lymphocytes were then resuspended in 0·25 ml of PBS and incubated overnight in PBS at 4°C. The sheep erythrocytes were then added and rosetting performed as in Method A (*i.e.* with 1½ h incubation at 4°C).

*Statistical methods.*—The significance of the difference in the mean T-lymphocyte levels between the young and elderly groups and the elderly and cancer groups was determined using unpaired *t* tests.

The significance of the difference in mean T-lymphocyte levels within the one group using different rosetting methods was determined using paired *t* tests.

The inhibition caused by incubation in cancer serum was expressed as a percentage.

$$\text{Inhibition} = \frac{\%\text{E rosettes after incubation in autologous serum} - \%\text{E rosettes after incubation in cancer serum}}{\%\text{E rosettes after incubation in autologous serum}}$$

### RESULTS

The means ±s.d. of E-rosetting cells for the 3 groups using the 3 methods is set out in Table 1. The individual results for each subject are shown in the Figure.

### *Comparison of different rosetting methods*

(a) *For highest yield.*—The 3 groups of patients were tested using all 3 methods.

In the young normal group, overnight incubation in PBS (Method C) yielded

* These patient groups were chosen because we had previously found that most Stage I and Stage II breast-cancer patients had low T-lymphocyte levels and most had inhibitory sera (Whitehead *et al.*, 1976, 1977).

TABLE I.—*Comparison of Percentage E-rosetting Cells using 3 Different Methods*

| Group | No. | Mean age | Age range | Method | | |
|---|---|---|---|---|---|---|
| | | | | A | B | C |
| Young | 12 | 29±6 | 20–41 | 64±4·8* | 66±4·5 | 68±6·6 |
| Elderly | 20 | 62±13 | 44–87 | 54±4·7 | 58±2·4 | 63±3·2 |
| Cancer | 20 | 64±12 | 44–86 | 45±7·9 | 54±2·9 | 60±2·7 |

* Mean±s.d.

FIG.—Comparison of estimates of T-cell levels obtained in young people, elderly people and breast cancer patients using 3 different rosetting techniques. ● young; ▲ age-matched; ■ cancer.

slightly higher levels of T lymphocytes than did Method A ($P<0{\cdot}05$).

In both the cancer group and the elderly normal group both Methods B and C yielded significantly higher levels of T lymphocytes than did Method A.

(b) *For demonstrating differences between the subject groups.*—When the young and elderly groups were compared it was found that Methods A and B yielded highly significant differences between the 2 groups ($P<0{\cdot}001$). Using Method C the difference between the 2 groups was less significant ($P<0{\cdot}02$).

Methods A and B yielded highly significant differences ($P<0{\cdot}001$) between the elderly group and breast cancer group. Using Method C the differences between the 2 groups just reached significance.

*Simulation of E-rosette depression using incubation in cancer serum*

The mean inhibition of E-rosette formation of normal lymphocytes by the 20 cancer sera was 27% when tested by our standard rosetting method (Method A) (Table II). This inhibition was not significant if the rosettes were incubated overnight at 4°C before counting (mean inhibition 5%) or if the lymphocytes were incubated overnight at 4°C in PBS before rosetting (mean inhibition 1%).

TABLE II.—*Inhibition of E-rosette Formation by Cancer Serum*

| Method | % Rosette formation | | % Inhibition |
|---|---|---|---|
| | Control | Cancer serum | |
| 1 | 63±4 | 46±5 | 27±8 |
| 2 | 64±4 | 61±3 | 5±7 |
| 3 | 66±3 | 65±3 | 1 |

The mean inhibition by sera from the age-matched control group was 20%. This inhibition was also removed by overnight incubation.

### DISCUSSION

The results of this investigation provide an explanation for the differing levels of T lymphocytes in breast-cancer patients reported by different groups. Stjernswärd *et al.* (1972) and Nemoto *et al.* (1974) found no significant difference between the levels of E-rosetting cells in breast-cancer patients and normal controls. By contrast, both Keller *et al.* (1976) and ourselves (Whitehead *et al.*, 1976) reported that the percentage of E-rosetting cells was significantly depressed in breast-cancer patients when compared with age-matched controls. Stjernswärd *et al.* (1972) used a rosetting technique which included overnight incubation before counting, a procedure which we have found to be least satisfactory for demonstrating depressed E-rosetting cell levels. Nemoto *et al.* (1974) isolated lymphocytes by passage of leucocyte-rich plasma through nylon-wool columns, a procedure which is reported to bias T/B lymphocyte ratios in favour of T lymphocytes, and possibly also leads to the selective loss of a subpopulation of T lymphocytes (WHO/IARC Workshop, 1974). Keller *et al.* (1976) used a short incubation period (60 min at 4°C) and found a similar level of depression to that found in our study. Depressed levels of "active" rosettes have been described by Wybran and Fudenberg (1973). However, their methods were not included in this study, as they only detect a subpopulation of E-rosetting cells and are thus difficult to correlate with reports using techniques that detect "total" E rosetting.

There is a similar situation in relation to studies of the effect of age on E-rosetting cell levels. In this study, the greatest difference between E-rosetting cell levels in the elderly control group and young control group was shown using a short incubation period ($1\frac{1}{2}$ h) at 4°C. After overnight incubation, the levels of E rosettes in old people increased and the differences between the two groups decreased to values similar to those reported by Augener *et al.* (1974). Other investigators have reported smaller (Smith *et al.*, 1974) and larger differences (Carosella *et al.*, 1974) between these 2 groups, but the rosetting methods used have been different from those used in this study.

In this study we have found that the levels of E-rosetting cells in both the cancer patients tested and the elderly patients increased significantly and approached the levels found in young subjects, after overnight incubation (Method C). In contrast, the levels of E-rosetting cells in the young people only increased slightly after overnight incubation, indicating that the short incubation period is detecting most of the T lymphocytes in the young people, but only a proportion of T lymphocytes in the elderly subjects and cancer patients. These results explain the differences between the results reported by Stjernswärd *et al.* (1972) and those reported by Keller *et al.* (1976) and us (Whitehead *et al.*, 1976).

We have previously found that incubation of normal lymphocytes in sera from breast-cancer patients or patients with chronic benign disease, causes a depression in the number of E-rosetting cells detectable using our standard method of rosette formation (Whitehead *et al.*, 1977 and unpublished). In this study we have used inhibition by cancer sera and sera from elderly controls as a model to study the mechanism by which E-rosetting levels are increased by overnight incubation. Although depressed levels of E-rosetting cells were found using a short incubation period ($1\frac{1}{2}$ h) this depression was spontaneously removed by overnight incubation, either after rosette formation, or in PBS before rosette formation. These results simulate those found with the breast-cancer group and elderly group and confirm that overnight incubation is sufficient to increase significantly the level of E-rosetting cells. These observations suggest that there is a factor on the surface of E-rosetting cells of breast-cancer patients and normal elderly controls (or patients with chronic benign disease) which dissociates from the lymphocyte surface on overnight incubation in PBS. The relatively high levels of E-rosetting cells found in breast-cancer patients and elderly controls after rosette formation in the presence of foetal calf serum (Method B) may be due to an increased rate of dissociation of the factor from the lymphocyte surface under these conditions. The depression of E-rosetting cell levels and the presence of an inhibitory factor in the sera are not believed to be cancer specific for the following reasons:

(a) Not all cancer patients show depressed levels of E-rosetting cells or have inhibitory sera (Whitehead *et al.*, 1976, Whitehead *et al.*, 1977).

(b) Similar levels of depression are found in aged patients and patients with active benign disease (*e.g.* chronic osteomyelitis and ulcerative colitis).

(c) Other workers have found depressed levels of E-rosetting cells and a serum inhibitory factor in patients with acute viral hepatitis (Chisari and Edgington, 1975). These findings suggest that the factor causing the inhibition is a product of a tissue breakdown process such as would be found in all these instances.

This work was supported by a grant from the Cancer Research Campaign. We acknowledge with gratitude the assistance of Mr C. Teasdale and Miss G. Richardson in obtaining many of the blood samples and thank Mr R. G. Newcombe of the Department of Medical Statistics for statistical analysis of the data.

### REFERENCES

ALEXOPOULOS, C. & BABITIS, P. (1976) Age Dependence of T-lymphocytes. *Lancet*, i, 426.

AUGENER, W., COHNEN, G. & REUTER, A. (1974) Decrease of T-lymphocytes during Ageing. *Lancet*, i, 1164.

CAROSELLA, E. D., MOCHANKO, K. & BRAUM, M. (1974) Rosette Forming T Cells in Human Peripheral Blood at Different Ages. *Cell. Immun.*, **12**, 323.

CHISARI, F. V. & EDGINGTON, T. S. (1975) Lymphocyte E-rosette Inhibitory Factor: A Regulatory Serum Lipoprotein. *J. exp. Med.*, **142**, 1092.

KELLER, S. E., IOACHIM, H. L., PEARSE, T. & SILETTI, D. M. (1976) Decreased T-lymphocytes in Patients with Mammary Cancer. *Am. J. clin. Path.*, **65**, 445.

NEMOTO, T., HAN, T., MINOWADA, J., ANGKUR, V., CHAMBERLAIN, A. & DAO, T. L. (1974) Cell-mediated Immune Status of Breast Cancer Patients: Evaluation by Skin Tests, Lymphocyte Stimulation and Counts of Rosette-forming Cells. *J. natn. Cancer Inst.*, **53**, 641.

SMITH, M. A., EVANS, J. & STEEL, C. M. (1974) Age-related Variation in Proportion of Circulating T Cells. *Lancet*, ii, 922.

STJERNSWÄRD, J., JONDAL, M., VANKY, F., WIGZELL, H. & SEALY, R. (1972) Lymphopenia and Change in Distribution of Tumour B and T Lymphocytes in Peripheral Blood Induced by Irradiation for Mammary Carcinoma. *Lancet*, **i**, 1352.

TEASDALE, C., THATCHER, J., WHITEHEAD, R. H., CHARE, M. J. B. & HUGHES, L. E. (1976) Age dependence of T-lymphocytes. *Lancet*, i, 1410.

WHITEHEAD, R. H., THATCHER, J., TEASDALE, C., ROBERTS, G. P. & HUGHES, L. E. (1976) T and B Lymphocytes in Breast Cancer. Stage Relationship and Abrogation of T-lymphocyte Depression by Enzyme Treatment *In vitro*. *Lancet*, i, 330.

WHITEHEAD, R. H., ROBERTS, G. P., THATCHER, J., TEASDALE, C. & HUGHES, L. E. (1977) Masking of Receptors for Sheep Erythrocytes on Human T-lymphocytes by Sera from Breast Cancer Patients. *J. natn. Cancer Inst.*, **58**, 1573.

WHO/IARC WORKSHOP ON HUMAN T AND B CELLS (1974) Identification, Enumeration and Isolation of B and T Lymphocytes from Human Peripheral Blood. *Scand. J. Immun.*, **3**, 521.

WYBRAN, J. & FUDENBERG, H. H. (1973) Thymus-derived Rosette Forming Cells. *New Engl. J. Med.*, **288**, 1072.